# Healthcare-Based Multimodal Recovery Prediction for the Warfighter: A Retrospective Study

**DOI:** 10.1093/milmed/usag199

**Published:** 2026-08-06

**Authors:** Isabel S Smokelin, Rebecca L Spirgel, Miriam Cha, Vesela P Kovacheva, Kajal T Claypool

**Affiliations:** Bioanalytics Systems and Technologies, Lincoln Laboratory, Massachusetts Institute of Technology, Lexington, MA 02421-6426, United States; Bioanalytics Systems and Technologies, Lincoln Laboratory, Massachusetts Institute of Technology, Lexington, MA 02421-6426, United States; Artificial Intelligence Technology, Lincoln Laboratory, Massachusetts Institute of Technology, Lexington, MA 02421-6426, United States; Department of Anesthesiology, Perioperative and Pain Management, Brigham and Women’s Hospital, Boston, MA 02115, United States; Bioanalytics Systems and Technologies, Lincoln Laboratory, Massachusetts Institute of Technology, Lexington, MA 02421-6426, United States; Department of Biomedical Informatics, Harvard Medical School, Boston, MA 02115, United States

## Abstract

**Introduction:**

In Roles 3 and 4 military treatment facilities and civilian hospitals, medics, unit commanders, and clinicians require knowledge of warfighter recovery following injury or surgery to aid operational planning, improve readiness and conduct resource allocation. In this study we developed a machine learning model using pre- and intraoperative patient surgical data from electronic health records to predict short-term recovery outcomes following surgery and to derive insights into clinical factors driving recovery.

**Materials and Methods:**

We used the INSPIRE (INformative Surgical Patient dataset for Innovative Research Environment) dataset, a publicly available, deidentified dataset from PhysioNet containing perioperative data from over 131,000 surgical procedures performed from 2011 to 2020 in South Korea to conduct our study. Data were originally collected under Institutional Review Board at Seoul National University Hospital and released publicly by their Institutional Data Review Board. Data contain patient demographics, surgical metadata, preoperative laboratory values, and pre-, post- and intraoperative vital signs. We defined recovery outcome as the time from the end of operation to discharge, which we binned into 3 categories: recovery in greater than 24 hours, recovery in 24 hours or less, or admission to the intensive care unit (ICU). We built several machine learning models (logistic regression, random forest and XGBoost) to predict our multiclass recovery outcome. Models were optimized with a 10-fold cross-validation procedure and evaluated with a custom area under the curve (AUC) metric where the predicted and true labels were one-hot encoded and a one-vs.-rest approach was used in calculation. We applied SHapley Additive Explanations to the features in the training data to determine which ones contributed most heavily to model predictions.

**Results:**

The best performing model was an XGBoost classifier, which reported a micro-AUC (samples given equal weight) of 0.948 and macro-AUC (classes given equal weight) of 0.815 on the 10% held out validation data. We found that intraoperative vital signs and department of procedure were the best indicators of recovery (>24 hours, ≤24 hours, or ICU).

**Conclusions:**

We developed an accurate model predicting time to recovery following surgery. Knowledge of important recovery predictive factors, such as intraoperative vital signs, provide crucial information to clinicians and army medics to inform recovery and potentially return to duty and required readiness levels. In the future, we aim to replicate this work in a clinical cohort in the United States primarily focused on a military population.

## INTRODUCTION

Medics, unit commanders, and clinicians are often faced with making critical decisions regarding injured warfighters from potential evacuation in Role 1 or 2 environments, to disposition planning and hospital discharge in Role 3 and 4 environments. In addition to decisions on recovery actions post injury or surgery, knowledge of medical factors that contribute to warfighter recovery in Role 3 and 4 environments can aid resource allocation and operational planning in Military Treatment Facilities (MTF). In the civilian sphere, hospitals often experience discharge delays, high census, and operational bottlenecks, which may cause decreased quality of care for patients and put pressure on the hospital system and staff. Following completion of a surgical procedure and the discontinuation of anesthesia in a Role 4 hospital, a patient is typically transferred to the post-anesthesia care unit (PACU), where they stay until they are stabilized for transfer to a ward, step-down unit, intensive care unit (ICU), or discharge home (for ambulatory cases). However, in some critical care cases and complex surgeries, patients can be transferred directly to the ICU instead of the PACU. Predictive models have been developed in healthcare settings to allow physicians and hospitals to predict and understand patient post-surgery outcomes: namely *mortality* post-surgery,^1-3^ and recovery defined as the length of stay in the PACU. In the civilian sphere, Chen et al. developed a fusion model of a deep neural network combined with Bidirectional Encoder Representation from Transformers to predict 30-day in-hospital postoperative mortality, and Lee et al. created an interpretable generalized additive neural network to predict postoperative in-hospital mortality.^2^^,^^3^ Other studies, such as those out of the University of California San Diego in 2017 and the University of Texas Southwestern in 2019, have built predictive models using preoperative data to identify patients at risk of extended stay (**≥**75th percentile of stay time) in the PACU following outpatient surgery.^4^^,^^5^ More recent work has developed models using preoperative data to predict PACU stay of greater than 4 hours for outpatient surgery, and ensemble models to predict if a surgery will be completed by the close of the operating room and if the patient will be discharged from the PACU before the close of the unit at the end of the day.^6^^,^^7^

Despite advancements in the field predicting various outcomes relating to mortality, and PACU stay time, fewer studies have framed postoperative recovery as a combined “disposition + discharge-timing” problem using both preoperative and intraoperative variables in a single multiclass model. Recovery can be a complex process, and in addition to the nature of the trauma, it depends on an individual’s medical history, current state of health, and genetics. In this study, recovery was defined as the time from the end of operation until discharge from the hospital and was categorized into 3 immediate, short-term outcomes: recovery in 24 hours or less, recovery in greater than 24 hours, and patients who were sent to the ICU after surgery. We posit that the inclusion of pre- and intraoperative data provides improved predictive power for patient recovery compared to solely preoperative data. Toward that end, we present an explainable machine learning model trained on a publicly available deidentified dataset containing preoperative and intraoperative data to accurately predict short-term recovery outcomes following surgery. In addition to delivering this accurate model across 3 recovery classes, we also derive insights into clinical factors driving recovery. Our results highlight that intraoperative vital signs and surgical specialty department of admission were the best indicators of recovery (>24 hours, ≤24 hours, or ICU). Predicting recovery in civilian hospitals and Role 4 MTF could potentially be a powerful tool to aid resource planning and rehabilitative care.

## METHODS

### Study Population

To develop our recovery model, we used the INSPIRE (INformative Surgical Patient dataset for Innovative Research Environment) dataset version 1.2, which contains perioperative data from 131,109 surgical procedures performed from 2011 to 2020 in South Korea.^8-10^ The study was approved under the Institutional Review Board at Seoul National University Hospital (SNUH, IRB No. H-2210-078-1368), and its data were publicly released under DRB No. BD-R-2022-11-02, after it was determined that the data were appropriately deidentified. The INSPIRE data set was originally derived from parent project VitalDB, a surgical vital signs database,^11^^,^^12^ and is publicly available, anonymized, and contains surgical procedure type, diagnoses, medications, intraoperative and preoperative vital signs, and laboratory data. Data were recorded before, during, and after surgery, and readings were anonymized by replacing values within each variable’s 2.5th-97.5th percentile range with rounded values at 5% intervals; values outside this range were replaced with the 2.5th or 97.5th percentile value to protect patient privacy.^8^ There was less than 0.002 percent reidentification risk of the entire data set in the reidentification analysis, thus assuring a high level of data security.^8^

### Defining Recovery

Our primary outcome was a 3-category variable “recovery” defined as recovery time in 24 hours or less, recovery time in greater than 24 hours, or postoperative admission to the ICU. These classes serve as the truth labels for the model predictions. These categories were chosen based on their clinical relevance and applicability to military medicine, a ≤24-hour discharge approximates ambulatory/short-stay recovery, >24-hour reflects inpatient recovery, and ICU admission represents a higher-acuity postoperative trajectory. Recovery time was calculated as the time from end of operation until discharge.

### Data Preprocessing and Feature Engineering

**Figure 1.**
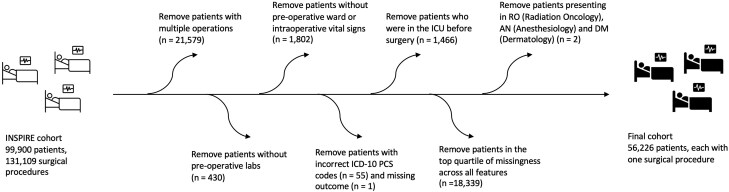
Preprocessing steps applied to down-select patients before model development.

We merged data from the original INSPIRE tables: operations (surgical metadata and patient demographic information), laboratory results (preoperative laboratory values), ward vital signs (pre- and postoperative vital signs), and vital signs (intraoperative vital signs), which gave us 99,000 patients and 131,109 surgical procedures (Figure 1). We then filtered the operations table to remove any patients with multiple surgeries, patients missing preoperative laboratory values, and patients missing preoperative ward and surgical vital signs. We also filtered laboratory values so that only the most recent of each laboratory value preceding the surgery remained, to reduce model complexity. We limited ward vital signs and vital signs to clinician-identified relevant features, which we aggregated as maximum, minimum, and/or mean value present before surgery, or in the intraoperative window (per the original vital signs data table). Subsequently, we removed patients missing recovery outcome or mapped to outdated ICD-10 procedural codes. Lastly, we omitted patients present in the ICU before surgery, as they were likely significantly less healthy and more unstable than the rest of the cohort, and could skew the model because of longer recovery times or eventual death.

After initial quality control, we one-hot encoded categorical features and subsequently removed patients within the top quartile of missingness across all features. We removed 26 non-Boolean clinical features with greater than 55 percent missingness. Our last quality control step included omitting patients presenting in surgical specialty departments who represented either 1 or 0 instances after previous preprocessing steps and would not be able to split evenly in model training. Lastly, based on clinical expertise, we imputed any remaining missing Boolean variables to false, and replaced missing continuous variables with the median of the feature. There were no missing categorical variables. Our final cohort included 56,226 patients, each with one surgical procedure (Figure 1). The flow of cohort selection can be seen in Figure 1.

### Model Development and Evaluation

We first split the data into 90 percent training + testing and 10 percent held-out validation using sci-kit learn’s train_test_split with shuffling to randomize the order of the data, and a fixed random seed to ensure reproducibility. The 90% training + testing data were used for model development, cross-validation and hyperparameter tuning, and the 10% held-out validation data were never used in this process, but instead to evaluate the ability of a model to generalize to unseen data. We developed 3 model types to predict our multiclass recovery outcome: a logistic regression, a random forest classifier, and an XGBoost classifier from the scikit-learn package. Using the 90% training + testing data, we optimized each model type with a 10-fold cross-validation using scikit-learn’s GridSearchCV to examine all permutations of hyperparameters in each hyperparameter grid. We chose 10 cross-validation folds to balance performance across folds and applied scikit-learn’s Pipeline object and StratifiedKFold cross-validator to examine model performance across different folds in the training data. For each fold, the Pipeline fit a standard scaler to the 9 training data folds, which was then applied to the single remaining test data fold. Folds were balanced using the stratified *k*-fold object to ensure representative numbers of classes in each fold.

We evaluated the models using a custom scoring metric inspired by scikit-learn’s literature, where we used a one-vs.-rest approach to calculate area under the curve (AUC).^13^ In this method, we one-hot encoded the truth and predicted labels before calculating true and false positive rates and AUC.^13^ This approach allowed us to understand the model performance across the entire dataset when each class was given an equal weight (macro-AUC), and when each data instance was given an equal weight (micro-AUC).

For hyperparameter optimization, we evaluated the logistic regression using both a multinomial and one-vs.-rest approach for the multiclass evaluation. For the random forest, we implemented a hyperparameter search examining 27 permutations across number of estimators (trees), maximum tree depth, and maximum features. For the XGBoost, we tested 18 permutations to optimize number of estimators, maximum depth, alpha value, learning rate, and subsampling, and employed a multi-softmax objective. The range of hyperparameters selected was identified using XGBoost literature^14^ and prior knowledge working with EHR data. For each model type (random forest, logistic regression, XGBoost), we used the hyperparameters that produced the highest macro-AUC on test folds from the cross-validation procedure to retrain the models with entire 90 training + testing data, before evaluating the retrained model on the 10 percent held-out data. We optimized the XGBoost model with 500 estimators (trees), a maximum depth of 6, alpha value of 2, learning rate of 0.1, and subsampling ratio of 0.5. We tuned the random forest model with 500 estimators and a maximum depth of 44, and the logistic regression with one-vs.-rest. After optimizing the XGBoost model, random forest model, and logistic regression model independently, we then identified the model that could best generalize to unseen data (produced the highest macro-AUC on the 10% held-out data). We then applied Shapley Additive Explanations to evaluate the features in the training data that contributed most heavily to this model’s prediction.^15^^,^^16^

## RESULTS

### Cohort Characteristics

A cohort of 56,226 patients (Figure 1), each with one surgery, was used for analysis. Table 1 displays our cohort characteristics, stratified by the 3 outcome classes (>24 hours: *n* = 47,882 [85.16%]; ≤24 hours: *n* = 1,449 [2.58%]; and ICU: *n* = 6,895 [12.26%]). Our selected cohort contained patients admitted to 1 of 12 surgical departments. The 5 largest categories across the overall cohort were general surgery (35.75%), obstetrics and gynecology (11.34%), orthopedic surgery (10.36%), neurosurgery (9.90%), and otolaryngology (9.45%) (Table 1). The overall cohort was 43.42% male and 56.58% female (Table 1). General anesthesia was administered to 97.77% of the cohort, and neuraxial (1.80%), monitored anesthesia care [MAC] (0.39%), and regional (0.04%) anesthesia were given to the remaining population (Table 1). Age remained binned into 5-year intervals, as predetermined for anonymity by the original data source, with 54 years as the mean age of the overall cohort (Table 1). Selected demographic and operational information, as well as characteristics found to contribute importance to model predictions from the Shapley value analysis are shown in Table 1, which details the statistical stratification across the 3 predictive classes and overall cohort. We found that the intraoperative mean arterial systolic and diastolic blood pressures for both the >24-hour (systolic: 114.74 mm Hg, diastolic: 61.45 mm Hg) and **≤**24 recovery class (systolic: 114.21 mm Hg, diastolic: 60.80 mm Hg) exceeded those of patients in the ICU class (systolic: 111.59 mm Hg, diastolic: 58.14 mm Hg) (Table 1). Additionally, 28.96% of the patients in the ICU class received intraoperative transfused red blood cells, compared to only 3.96% from the >24-hour class and 0.14% from the **≤**24-hour class (Table 1). Most patients whose medical records had an ICD-10 code relating to the female reproductive system fell into the **≤**24-hour recovery class (35.68%), compared to >24 hour (6.03%) and ICU (0.39%) (Table 1). Lastly, the mean recovery time for the ICU class (280.63 hours) exceeded that of both the >24-hour class (122.73 hours), and the **≤**24-hour class (12.58 hours). Expanded tables including all variables can be viewed in the online supplementary material (Tables S1-S3), as well as additional visualizations of overall cohort characteristics (Figure S1).

**Table 1.** Cohort Characteristics

Characteristic	Overall	>24 hours	≤24 hours	ICU
	***N* = 56,226 (95% CI)** ^a^	***N* = 47,882 (95% CI)** ^a^	***N* = 1,449 (95% CI)** ^a^	***N* = 6,895 (95% CI)** ^a^
Anesthesia type				
General anesthesia	97.77% (98–98)	97.64% (97–98)	96.14% (95–97)	99.00% (99–99)
Monitored anesthesia care (MAC)	0.39% (0.34–0.45)	0.32% (0.28–0.38)	3.73% (2.8–4.9)	0.16% (0.08–0.29)
Neuraxial anesthesia	1.80% (1.7–1.9)	2.00% (1.9–2.1)	0.14% (0.02–0.56)	0.80% (0.61–1.0)
Regional anesthesia	0.04% (0.03–0.06)	0.04% (0.02–0.06)	0.00% (0.00–0.33)	0.04% (0.01–0.14)
Surgical specialty department of admission				
Cardio-thoracic surgery	8.14% (7.9–8.4)	4.35% (4.2–4.5)	0.76% (0.40–1.4)	35.97% (35–37)
General surgery	35.75% (35–36)	38.38% (38–39)	6.21% (5.0–7.6)	23.70% (23–25)
Internal medicine	0.08% (0.06–0.11)	0.09% (0.07–0.13)	0.00% (0.00–0.33)	0.01% (0.00–0.09)
Neurosurgery	9.90% (9.7–10)	7.02% (6.8–7.2)	0.48% (0.21–1.0)	31.95% (31–33)
Obstetrics & gynecology	11.34% (11–12)	11.97% (12–12)	36.78% (34–39)	1.61% (1.3–1.9)
Oto-laryngology	9.45% (9.2–9.7)	10.29% (10–11)	18.50% (17–21)	1.70% (1.4–2.0)
Orthopedic surgery	10.36% (10–11)	11.62% (11–12)	1.59% (1.0–2.4)	3.44% (3.0–3.9)
Ophthalmology	2.93% (2.8–3.1)	2.72% (2.6–2.9)	23.33% (21–26)	0.07% (0.03–0.18)
Pediatrics	0.04% (0.02–0.06)	0.04% (0.03–0.07)	0.00% (0.00–0.33)	0.00% (0.00–0.07)
Plastic surgery	2.91% (2.8–3.1)	2.99% (2.8–3.1)	11.25% (9.7–13)	0.62% (0.46–0.85)
Radiology	0.37% (0.33–0.43)	0.41% (0.35–0.47)	0.00% (0.00–0.33)	0.23% (0.14–0.39)
Urology	8.73% (8.5–9.0)	10.12% (9.9–10)	1.10% (0.65–1.8)	0.70% (0.52–0.93)
Intraoperative vital signs				
Transfused red blood cell	6.93% (6.7–7.1)	3.96% (3.8–4.1)	0.14% (0.02–0.56)	28.96% (28–30)
Infused volume of plasma solution	49.94% (50–50)	46.32% (46–47)	17.46% (16–20)	81.89% (81–83)
Injected dose of propofol	44.54% (44–45)	47.79% (47–48)	65.70% (63–68)	17.48% (17–18)
Mean arterial systolic blood pressure, mm Hg	114.34, 7.77 (114, 114)	114.74, 7.08 (115, 115)	114.21, 2.93 (114, 114)	111.59, 11.55 (111, 112)
Mean arterial diastolic blood pressure, mm Hg	61.03, 5.18 (61, 61)	61.45, 4.80 (61, 61)	60.80, 1.65 (61, 61)	58.14, 6.93 (58, 58)
Mean arterial mean blood pressure, mm Hg	78.19, 7.60 (78, 78)	78.63, 7.35 (79, 79)	78.06, 4.40 (78, 78)	75.17, 9.02 (75, 75)
Mean non-invasive systolic blood pressure, mm Hg	120.23, 16.33 (120, 120)	119.82, 15.80 (120, 120)	116.82, 15.74 (116, 118)	123.79, 19.28 (123, 124)
Mean body temperature, °C	34.93, 2.08 (35, 35)	34.96, 2.08 (35, 35)	34.56, 2.44 (34, 35)	34.79, 2.03 (35, 35)
Maximum body temperature, °C	36.11, 0.93 (36, 36)	36.10, 0.91 (36, 36)	35.71, 1.57 (36, 36)	36.24, 0.79 (36, 36)
Minimum body temperature, °C	31.32, 5.78 (31, 31)	31.39, 5.79 (31, 31)	31.95, 5.37 (32, 32)	30.73, 5.81 (31, 31)
Mean tidal volume, mL	430.51, 73.40 (430, 431)	432.32, 73.06 (432, 433)	417.96, 68.57 (414, 421)	420.53, 75.66 (419, 422)
Mean peripheral oxygen saturation, percentage	99.11, 1.04 (99, 99)	99.11, 1.03 (99, 99)	98.79, 1.38 (99, 99)	99.22, 0.96 (99, 99)
Mean heart rate/min	70.32, 11.04 (70, 70)	70.04, 10.81 (70, 70)	73.16, 11.28 (73, 74)	71.63, 12.34 (71, 72)
Mean bispectral index	40.74, 5.34 (41, 41)	40.85, 5.39 (41, 41)	40.97, 5.51 (41, 41)	39.94, 4.87 (40, 40)
Sex—male	43.42% (43–44)	41.79% (41–42)	32.51% (30–35)	57.01% (56–58)
Age of the patient on the operation date, years	54.00, 15.73 (54, 54)	53.49, 15.63 (53, 54)	45.66, 15.98 (45, 46)	59.34, 14.95 (59, 60)
Recovery time, hours^b^	139.26, 188.09 (138, 141)	122.73, 155.74 (121, 124)	12.58, 2.87 (12, 13)	280.63, 307.94 (273, 288)
ICD-10 Code—female reproductive system	6.10% (5.9–6.3)	6.03% (5.8–6.2)	35.68% (33–38)	0.39% (0.26–0.58)
Preoperative maximum body temperature, °C	36.77, 0.41 (37, 37)	36.75, 0.40 (37, 37)	36.53, 0.40 (37, 37)	36.94, 0.47 (37, 37)

Selected demographic and medical characteristics for overall cohort and stratified by 3 outcomes (>24 hours, ≤24 hours, and ICU).

Abbreviations: CI, confidence interval; ICU, intensive care unit; SD, standard deviation.

a Values are reported as the mean, SD (CI) for continuous variables, and as percentage (CI) for categorical and binary variables. 95% confidence intervals shown in parentheses.

b Recovery time is defined as the time in hours from the end of operation to discharge.

### Modeling

Table 2 reports the performance metrics, micro- and macro-AUCs for the 3 models: logistic regression, random forest, and XGBoost. Of the 3 model types, the optimized XGBoost model generalized best to unseen data (micro-AUC: 0.948, macro-AUC: 0.815), compared to the random forest (micro-AUC: 0.946, macro-AUC: 0.811), and logistic regression (micro-AUC: 0.931, macro-AUC: 0.708) (Table 2). In each class, the XGBoost model reported validation (external held-out) AUCs of 0.789 (≤24 hours), 0.823 (>24 hours), and 0.834 (ICU). (Table 2). Across all models, the micro-AUC exceeded the comparative macro-AUC. Further, the accuracy of the XGBoost (0.93) on the validation held-out data exceeded that of the random forest (0.927) and logistic regression (0.908).

**Table 2.** Recovery Model Performance on INSPIRE Patient Data Across 3 Different Model Types (Logistic Regression, Random Forest, and XGBoost)

		Logistic regression	Random forest	XGBoost
Optimized hyperparameters		One-vs-rest	*n*_estimators: 500 max_depth: 44 max_features: None	*n*_estimators: 500 max_depth : 6 classes: 3 alpha: 2 learning rate: 0.1 subsample: 0.5 objective: multi: softmax
Training accuracy		0.908	0.999	0.997
Validation accuracy		0.908	0.927	0.93
Training AUC	≤24 hours	0.576	0.991	0.992
	>24 hours	0.744	0.998	0.99
	ICU	0.782	0.999	0.989
	Micro	0.931	0.999	0.997
	Macro	0.7	0.996	0.99
Validation AUC	≤24 hours	0.586	0.792	0.789
	>24 hours	0.75	0.816	0.823
	ICU	0.788	0.825	0.834
	Micro	0.931	0.946	0.948
	Macro	0.708	0.811	0.815

Training performance reported on 90% data. Validation performance reported on 10% held out data. Hyperparameters selected were those that yielded the best performance in hyperparameter optimization.

Abbreviations: AUC, area under the curve; ICU, intensive care unit.

### Explainability

**Figure 2.**
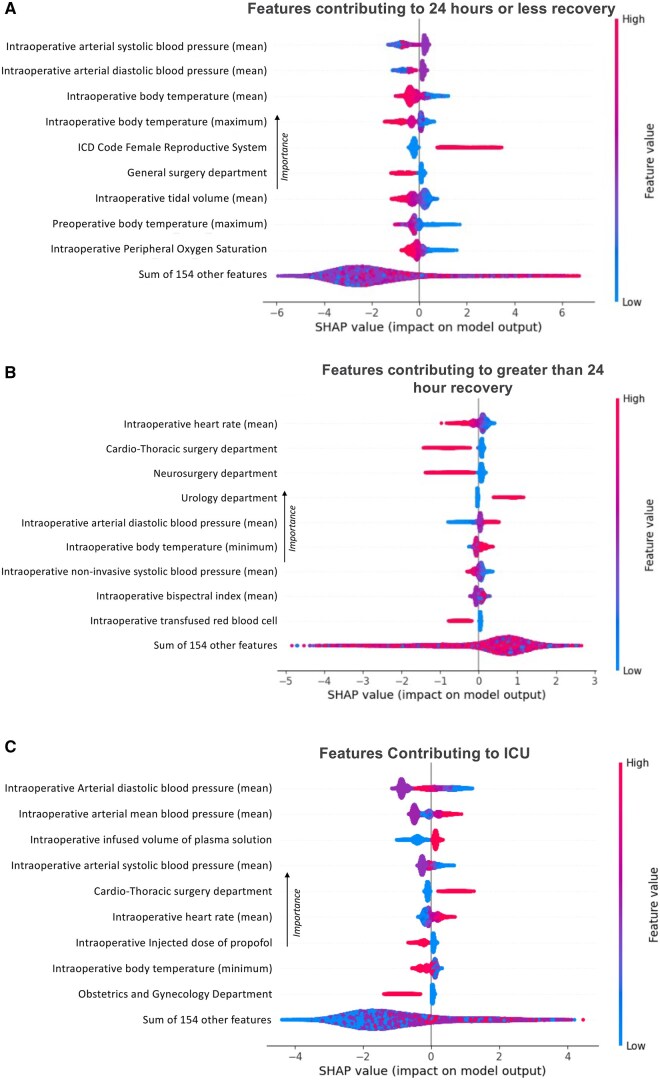
Shapley beeswarm plots reveal the clinical features present in the training data of the XGBoost model that most heavily contributed to the model predictions of the recovery outcome classes (A) ≤24 hours recovery, (B) >24 hours recovery, and (C) intensive care unit (ICU). Features listed higher on the plot’s *y*-axis have greater contribution toward model’s class prediction, and the shape of the feature distribution map across the *x*-axis reflects how the feature impacts the model output. Red color indicates higher values of a feature, and blue maps to lower feature values. Abbreviation: SHAP, SHapley additive exPlanations.

Figure 2 shows the results of the Shapley analysis for the 3 outcomes of interest (≤24 hours, >24 hours, and ICU). Features listed higher on a Shapley plot’s *y*-axis have a greater contribution toward the model’s class prediction, and the shape of the feature distribution map across the *x*-axis reflects the SHAP values, which reveal how each feature impacts the model output. Positive SHAP values contribute to the model’s prediction, whereas negative SHAP values detract from the prediction. Red color indicates higher values of a feature, and blue color maps to lower feature values. Each sub Figure 2A-C shows the top 9 features contributing toward the model’s prediction for each class in descending order of importance, as well as an aggregation of 154 other features. To put this in context, from Figure 2A we can infer that mean intraoperative arterial systolic blood pressure was the most important feature driving model prediction of ≤24 hour recovery, and the moderate blood pressures (purple points, positive SHAP axis), contributed to this class membership prediction while high blood pressures (red points) and low blood pressures (blue points), shown on the negative SHAP axis, detracted from this class prediction. More broadly, across all outcomes, vital signs and surgical specialty department of admission were key drivers for model prediction, which can be noted by their abundance in the *y*-axis labels of Figure 2A-C. Specifically, for the outcome ≤24-hours, intraoperative mean arterial systolic blood pressure and intraoperative mean arterial diastolic blood pressure, as well as mean and maximum intraoperative body temperatures drove class membership; this is evidenced by these 4 features’ labels appearing higher on the Shapley plot *y*-axis (Figure 2A). For the ICU outcome, arterial blood pressure-related features drove model prediction, with 3 of the 4 top features contributing to ICU class membership (Figure 2C). Mean intraoperative arterial diastolic blood pressure ranked as the most significant feature in the training data that drove the model’s prediction of ICU, as seen as the highest label on the *y*-axis in Figure 2C. Further, low intraoperative arterial diastolic blood pressure readings, shown as blue points on the positive SHAP axis, drove the ICU prediction, while more middle values (purple points, negative SHAP axis) detracted from ICU prediction (Figure 2C). This contrasted with the trends seen in the arterial blood pressures in Figure 2A, where the middle values (purple points, positive SHAP axis) contributed to ≤24-hour recovery, and high values (red points) and low values (blue points), both seen on the negative SHAP axis, detracted from this prediction. Lastly, Figure 2B revealed that admission to cardiothoracic surgery, neurosurgery, and urology were important factors in predicting membership to the >24-hour recovery class.

## DISCUSSION

We developed an accurate machine learning model to predict the duration of recovery from the end of surgery until discharge from the hospital using preoperative and intraoperative data from a publicly available dataset derived from the electronic health records of 56,226 patients from South Korea. We found that XGBoost performed the best on unseen data across the outcome classes with micro-AUC 0.948 and macro-AUC: 0.815.

The results of the recovery model reflect a combination of the inherent features of the data, as well as feature engineering and model-building choices. The XGBoost and Random Forest performed similarly, and both performed better than the logistic regression, indicating that more advanced machine learning models are better able to capture nonlinear relationships in data.

We compare our results with the work of Gabriel et al and Rupp et al, who have developed models to predict the length of stay in the PACU for patients undergoing outpatient procedures. Although Rupp et al. focused solely on developing a logistic regression model, Gabriel et al. developed several different linear and nonlinear machine learning models. Similar to our results, they determined that the random forest outperformed the logistic regression, highlighting the importance of developing a machine learning model complex enough to capture the relationships in EHR data.

Additionally, our finding that the ICU class mean recovery time (280.63 hours) exceeded the mean >24-hour class recovery time (122.73 hours), supports use of this model for operational planning and resource allocation. As we consider large scale combat operations where an understanding of how quickly individuals can be moved out of the ICU or MTF could have a sizeable impact on the MTF resource planning, these short-term immediate recovery bins can serve as a useful surrogate for hospital stay and resource needs. Further, these predictions can inform army medics and clinicians of individual readiness levels and potential return to duty.

Our explainability analysis revealed key medical factors driving the model’s predictions and may promote trust and adoption of the model in the military medical community to due increased understanding of the predictions. The results from our explainability Shapley value plots are consistent with known patterns in clinical practice, in addition, a few novel insights emerged about features associated with outcomes. It is not surprising that patients with abnormal vital signs require extended inpatient recovery or need critical care, as fever and low blood pressure are most likely because of severe or critical health conditions, which often correspond to ICU trajectory. Similarly, large and complex surgical procedures, such as cardiothoracic surgery may require going to the ICU, while surgeries relating to the female reproductive system may be localized and enable patient discharge home the same day, in ≤24 hours. Further, patients with normal blood pressure values and temperature are more likely to have lower acuity and, subsequently, ≤24 hours of hospital stay. In both the ICU and ≤24-hour recovery categories, we see some of the same features, such as blood pressure, appear as highly important, but their values impact the outcome inversely, highlighting how intraoperative vital signs can shed insight into recovery trajectory and the state of patient health. Tidal volume was associated with ≤24-hour recovery which is a novel insight that we recommend replicating in future studies. The Shapley plot for the >24-hour recovery revealed membership to the urology department supports prediction to this category.

Although our outcome definition differs from prior PACU-focused studies, surgical specialty was an important factor driving model prediction, highlighting this as a useful indicator for understanding recovery and potential return to service. Other models predicting PACU duration excluded intraoperative data as predictors, as they were aimed at forecasting before having knowledge during surgery to inform scheduling in the operating room. Although intraoperative data must be obtained in real time and thus cannot be known before scheduling a surgery, we found that intraoperative vital signs were highly important in predicting the recovery outcome. This information may be crucial for predicting discharge from the hospital in Roles 3 and 4 environments and still allow enough lead time from the point of collection to the point of discharge to which the model proves useful for unit commanders and army medics. Overall, examining the features driving the model prediction can support the use of this model as a decision support tool in military and civilian treatment facilities and promote trust in the model.

### Limitations and Future Work

Our analysis has several limitations which offer directions for future work. First, this dataset was anonymized by binning/rounding continuous values, and it may not reflect the complexity of real-time clinical data. Second, this was data from a single institution collected in the past and may not be representative of the diversity of our military and civilian cohorts, nor may it consider some recent improvements in clinical practice. Third, our work is limited to predicting recovery from single surgeries. Further, the missing data cannot be recovered or verified, thus our assumptions cannot be validated. Higher levels of missing data can introduce noise into the analysis, as they must be imputed before being sent to the recovery model. We attempted to mitigate this by dropping patients in the top quartile of missingness before filling missing variables, but this could have omitted clinical information. Our feature selection and engineering choices could potentially have consequences on model performance and/or represent a loss of information but ultimately were chosen based on clinical relevance and prior work. For example, we opted not to include duration of surgery as a variable because it is dependent on local clinical practices and available resources; in excluding it we prioritized preserving model generalizability and parsimony.

Another limitation is the significant class imbalance amongst recovery groups; the ≤24-hour recovery class represented just 2.6 percent of the cohort while the >24-hour recovery class represented 84.2 percent of the cohort. This class imbalance caused a performance gap between micro and macro-AUC values. As macro-AUC is calculated weighting each class equally, while micro-AUC gives each sample equal weight, micro-AUC is more heavily influenced by the group with the largest number of samples. Further, the best model can achieve only moderate discrimination on the ≤24-hour group (AUC 0.789) because of the smaller number of examples in this class. Next steps could include experimenting with techniques to further balance classes, such as random oversampling, but overall model performance reflects the actual distribution of the data.

In this study, we predict immediate short-term recovery outcomes based on time (≤24-hour, >24-hour), and acuity (ICU), which provides insight to clinicians and army medics about recovery trajectory for individuals recovering from surgery. Although this information is useful and may inform short-term operational readiness, it does not account for other factors such as clinical practice patterns and local treatment facility conditions, which may also impact the rate of medical resource use in both the ICU and PACU. For example, a short ICU stay may use fewer medical resources than a long stay in the PACU, so the chosen bins are not perfect surrogates for resource use. In future work it would be interesting to predict long-term resource needs by combining acuity and time bins to analyze low, medium, and high resource needs groups, as opposed to focusing on immediate short-term outcomes. Lastly, recovery after surgical procedures may not follow the same trajectory as recovery after military trauma, and therefore, the results from this study may not be fully relevant to warfighters. Future work should focus on the validation of this work in relevant real-world clinical cohorts from the United States and prospective studies to determine its true operational impact.

## CONCLUSION

We developed several machine learning models using preoperative and intraoperative data to predict postoperative recovery trajectory as a categorical outcome. Although all 3 models performed well, we found that the XGBoost model generalized best to unseen data, and that intraoperative vital signs and surgical department were key drivers of the model’s prediction. Future work will focus on model refinement, real-world validation, and evaluation in military-relevant populations and injury patterns. Clinical implementation of this model in civilian or Role 4 military hospitals could support staffing and resource allocation decisions and may improve operational planning and readiness.

## Supplementary Material

usag199_Supplementary_Data

## Data Availability

Data used in this article are publicly available from PhysioNet.

## INSTITUTIONAL CLEARANCE

Approved.

## References

[1] Choi B , Oh AR, Lee S-H, et al Prediction model for 30-day mortality after non-cardiac surgery using machine-learning techniques based on preoperative evaluation of electronic medical records. J Clin Med. 2022;11:6487. 10.3390/jcm11216487 36362715 PMC9659244

[2] Chen P-F , Chen L, Lin Y-K, et al Predicting postoperative mortality with deep neural networks and natural language processing: model development and validation. JMIR Med Inform. 2022;10:e38241. 10.2196/38241 35536634 PMC9131148

[3] Lee CK , Samad M, Hofer I, Cannesson M, Baldi P. Development and validation of an interpretable neural network for prediction of postoperative in-hospital mortality. NPJ Digit Med. 2021;4:8. 10.1038/s41746-020-00377-1 33420341 PMC7794438

[4] Elsharydah A , Walters DR, Somasundaram A, et al A preoperative predictive model for prolonged post-anaesthesia care unit stay after outpatient surgeries. J Perioper Pract. 2020;30:91–6. 10.1177/1750458919850377 31135281

[5] Gabriel RA , Waterman RS, Kim J, Ohno-Machado L. A predictive model for extended postanesthesia care unit length of stay in outpatient surgeries. Anesth Analg. 2017;124:1529–36. 10.1213/ANE.0000000000001827 28079580

[6] Rupp S , Ahrens E, Rudolph MI, et al Development and validation of an instrument to predict prolonged length of stay in the postanesthesia care unit following ambulatory surgery. Can J Anaesth. 2023;70:1939–49. 10.1007/s12630-023-02604-1 37957439

[7] Gabriel RA , Harjai B, Simpson S, Goldhaber N, Curran BP, Waterman RS. Machine learning-based models predicting outpatient surgery end time and recovery room discharge at an ambulatory surgery center. Anesth Analg. 2022;135:159–69. 10.1213/ANE.0000000000006015 35389380 PMC9172889

[8] Lim L , Lee H-C. INSPIRE, a publicly available research dataset for perioperative medicine (version 1.2). Published online 2023: RRID: SCR_007345. 10.13026/4evs-wq50 PMC11192876 38906912

[9] Lim L , Lee H, Jung C-W, et al INSPIRE, a publicly available research dataset for perioperative medicine. Sci Data. 2024;11:655. 10.1038/s41597-024-03517-4 38906912 PMC11192876

[10] Goldberger AL , Amaral LAN, Glass L, et al PhysioBank, PhysioToolkit, and PhysioNet: components of a new research resource for complex physiologic signals. *Circulation.* 2000;101:e215–20. 10.1161/01.CIR.101.23.e215 10851218

[11] Lee H-C , Park Y, Yoon SB, Yang SM, Park D, Jung C-W. VitalDB, a high-fidelity multi-parameter vital signs database in surgical patients. Sci Data. 2022;9:279. 10.1038/s41597-022-01411-5 35676300 PMC9178032

[12] Lee HC , Jung C-W. VitalDB, a high-fidelity multi-parameter vital signs database in surgical patients. Published online September 21, 2022: RRID: SCR_007345. 10.13026/czw8-9p62 PMC9178032 35676300

[13] scikit-learn developers. Multiclass receiver operating characteristic (ROC). https://scikit-learn.org/stable/auto_examples/model_selection/plot_roc.html

[14] xgboost developers. XGBoost Parameters. 2024. https://xgboost.readthedocs.io/en/stable/parameter.html

[15] Scott Lundberg. An introduction to explainable AI with Shapley values. 2018. https://shap.readthedocs.io/en/latest/example_notebooks/overviews/An%20introduction%20to%20explainable%20AI%20with%20Shapley%20values.html

[16] Lloyd Shapley. A value for n-person games. Published online 1953:307–317. 10.1515/9781400881970-018

