# Supplementary material for: Healthcare-Based Multimodal Recovery Prediction for the Warfighter: A Retrospective Study
Source: Mil Med. 2026 Aug 6;191(Suppl 1):631–40. doi: 10.1093/milmed/usag199 (PMC13445627; doi:10.1093/milmed/usag199)
Supplement: Supplementary file 1 [file usag199_supplementary_data.zip › usag199_Supplementary_Data/FigureS1_revised.pdf]

DISTRIBUTION STATEMENT A. Approved for public release. Distribution is unlimited.

This material is based upon work supported by the Department of the Air Force under Air Force Contract No. FA8702-15-D-0001 or FA8702-25-D-B002. Any opinions, findings, conclusions or recommendations expressed in this material are those of the author(s) and do not necessarily reflect the views of the Department of the Air Force.

© 2026 Massachusetts Institute of Technology.

Delivered to the U.S. Government with Unlimited Rights, as defined in DFARS Part 252.227-7013 or 7014 (Feb 2014). Notwithstanding any copyright notice, U.S. Government rights in this work are defined by DFARS 252.227-7013 or DFARS 252.227-7014 as detailed above. Use of this work other than as specifically authorized by the U.S. Government may violate any copyrights that exist in this work.

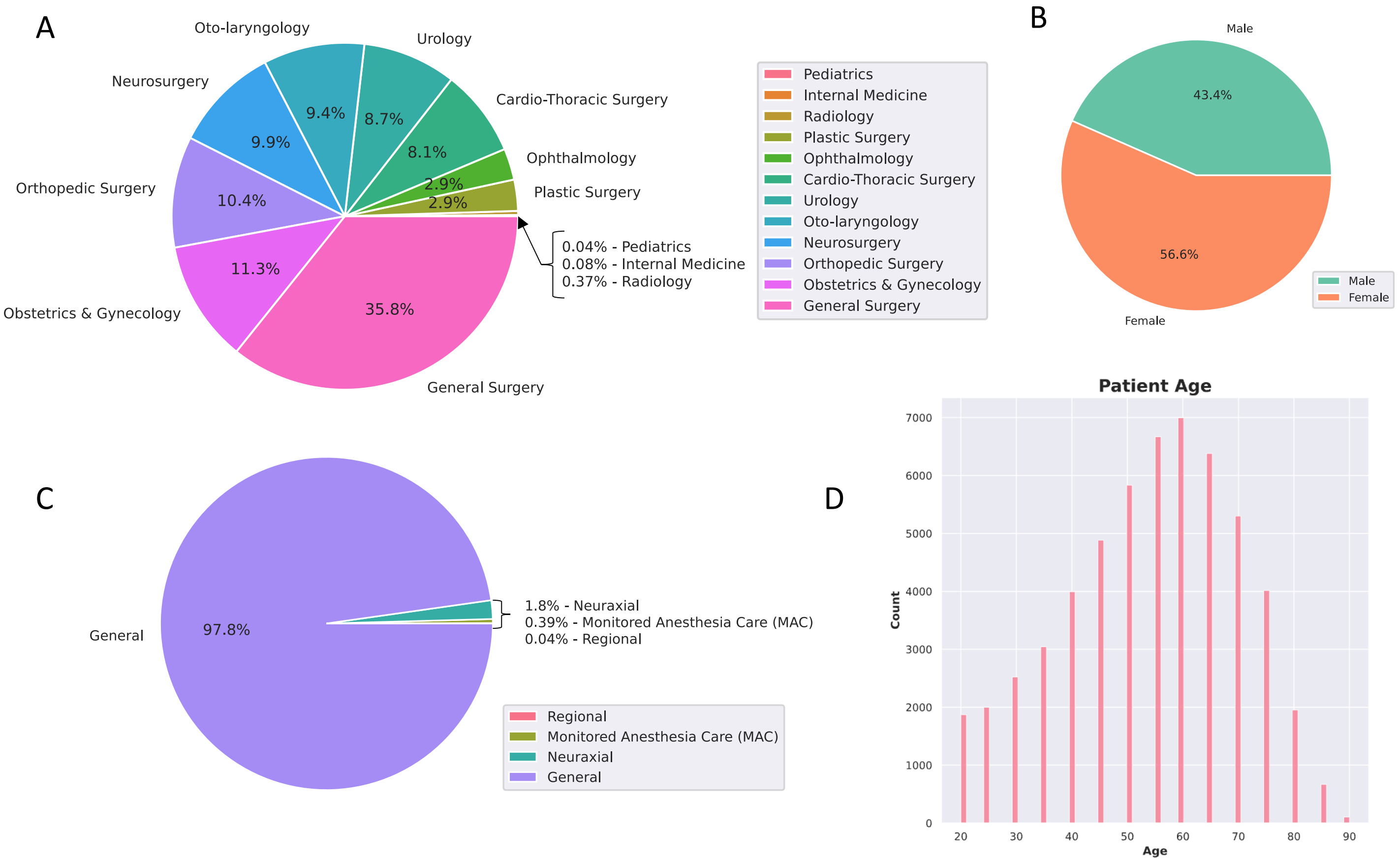

Figure S1. Demographic characteristics of overall cohort based on (a) surgical department of admission, (b) sex, (c) type of anesthesia administered, and (d) age, which remains binned based on original data source.
