# Supplementary material for: Healthcare-Based Multimodal Recovery Prediction for the Warfighter: A Retrospective Study
Source: Mil Med. 2026 Aug 6;191(Suppl 1):631–40. doi: 10.1093/milmed/usag199 (PMC13445627; doi:10.1093/milmed/usag199)
Supplement: Supplementary file 1 [file usag199_supplementary_data.zip › usag199_Supplementary_Data/Table_S1.docx]

| **Table S1. Medical and Surgical Metrics** | | | | |
| --- | --- | --- | --- | --- |
| **Characteristic** | **Overall**  N = 56,226 (**95% CI**)* | **> 24 hr**  N = 47,882 (**95% CI**)* | **≤ 24 hr**  N = 1,449 (**95% CI**)* | **ICU**  N = 6,895 (**95% CI**)* |
| **Surgical Specialty Department for Admission** |  |  |  |  |
| *Cardio-Thoracic Surgery* | 8.14% (7.9%, 8.4%) | 4.35% (4.2%, 4.5%) | 0.76% (0.40%, 1.4%) | 35.97% (35%, 37%) |
| *General Surgery* | 35.75% (35%, 36%) | 38.38% (38%, 39%) | 6.21% (5.0%, 7.6%) | 23.70% (23%, 25%) |
| *Internal Medicine* | 0.08% (0.06%, 0.11%) | 0.09% (0.07%, 0.13%) | 0.00% (0.00%, 0.33%) | 0.01% (0.00%, 0.09%) |
| *Neurosurgery* | 9.90% (9.7%, 10%) | 7.02% (6.8%, 7.2%) | 0.48% (0.21%, 1.0%) | 31.95% (31%, 33%) |
| *Obstetrics & Gynecology* | 11.34% (11%, 12%) | 11.97% (12%, 12%) | 36.78% (34%, 39%) | 1.61% (1.3%, 1.9%) |
| *Oto-laryngology* | 9.45% (9.2%, 9.7%) | 10.29% (10%, 11%) | 18.50% (17%, 21%) | 1.70% (1.4%, 2.0%) |
| *Orthopedic Surgery* | 10.36% (10%, 11%) | 11.62% (11%, 12%) | 1.59% (1.0%, 2.4%) | 3.44% (3.0%, 3.9%) |
| *Ophthalmology* | 2.93% (2.8%, 3.1%) | 2.72% (2.6%, 2.9%) | 23.33% (21%, 26%) | 0.07% (0.03%, 0.18%) |
| *Pediatrics* | 0.04% (0.02%, 0.06%) | 0.04% (0.03%, 0.07%) | 0.00% (0.00%, 0.33%) | 0.00% (0.00%, 0.07%) |
| *Plastic Surgery* | 2.91% (2.8%, 3.1%) | 2.99% (2.8%, 3.1%) | 11.25% (9.7%, 13%) | 0.62% (0.46%, 0.85%) |
| *Radiology* | 0.37% (0.33%, 0.43%) | 0.41% (0.35%, 0.47%) | 0.00% (0.00%, 0.33%) | 0.23% (0.14%, 0.39%) |
| *Urology* | 8.73% (8.5%, 9.0%) | 10.12% (9.9%, 10%) | 1.10% (0.65%, 1.8%) | 0.70% (0.52%, 0.93%) |
| **Emergency of the Operation (binary)** | 7.46% (7.2%, 7.7%) | 6.82% (6.6%, 7.0%) | 3.66% (2.8%, 4.8%) | 12.72% (12%, 14%) |
| **Recovery time, hours**** | 139.26, 188.09 (138, 141) | 122.73, 155.74 (121, 124) | 12.58, 2.87 (12, 13) | 280.63, 307.94 (273, 288) |
| **Anesthesia Type** |  |  |  |  |
| *General* | 97.77% (98%, 98%) | 97.64% (97%, 98%) | 96.14% (95%, 97%) | 99.00% (99%, 99%) |
| *Monitored Anesthesia Care (MAC)* | 0.39% (0.34%, 0.45%) | 0.32% (0.28%, 0.38%) | 3.73% (2.8%, 4.9%) | 0.16% (0.08%, 0.29%) |
| *Neuraxial* | 1.80% (1.7%, 1.9%) | 2.00% (1.9%, 2.1%) | 0.14% (0.02%, 0.56%) | 0.80% (0.61%, 1.0%) |
| *Regional* | 0.04% (0.03%, 0.06%) | 0.04% (0.02%, 0.06%) | 0.00% (0.00%, 0.33%) | 0.04% (0.01%, 0.14%) |
| **ICD-10 Procedural Coding System Code** |  |  |  |  |
| *Medical & Surgical - Central Nervous System and Cranial Nerves* | 4.57% (4.4%, 4.7%) | 2.97% (2.8%, 3.1%) | 0.48% (0.21%, 1.0%) | 16.48% (16%, 17%) |
| *Medical & Surgical - Peripheral Nervous System* | 0.52% (0.46%, 0.58%) | 0.57% (0.50%, 0.64%) | 0.83% (0.45%, 1.5%) | 0.10% (0.04%, 0.22%) |
| *Medical & Surgical - Heart and Great Vessels* | 2.19% (2.1%, 2.3%) | 0.21% (0.17%, 0.25%) | 0.00% (0.00%, 0.33%) | 16.40% (16%, 17%) |
| *Medical & Surgical - Upper Arteries* | 2.32% (2.2%, 2.5%) | 2.12% (2.0%, 2.3%) | 0.48% (0.21%, 1.0%) | 4.13% (3.7%, 4.6%) |
| *Medical & Surgical - Lower Arteries* | 0.31% (0.27%, 0.36%) | 0.06% (0.04%, 0.08%) | 0.07% (0.00%, 0.45%) | 2.12% (1.8%, 2.5%) |
| *Medical & Surgical - Upper Veins* | 0.37% (0.32%, 0.43%) | 0.41% (0.36%, 0.47%) | 0.28% (0.09%, 0.76%) | 0.13% (0.06%, 0.26%) |
| *Medical & Surgical - Lower Veins* | 0.09% (0.07%, 0.12%) | 0.11% (0.08%, 0.14%) | 0.00% (0.00%, 0.33%) | 0.01% (0.00%, 0.09%) |
| *Medical & Surgical - Lymphatic and Hemic Systems* | 1.78% (1.7%, 1.9%) | 1.90% (1.8%, 2.0%) | 0.48% (0.21%, 1.0%) | 1.26% (1.0%, 1.6%) |
| *Medical & Surgical - Eye* | 2.88% (2.7%, 3.0%) | 2.66% (2.5%, 2.8%) | 23.26% (21%, 26%) | 0.13% (0.06%, 0.26%) |
| *Medical & Surgical - Ear, Nose, Sinus* | 10.62% (10%, 11%) | 11.38% (11%, 12%) | 15.25% (13%, 17%) | 4.41% (3.9%, 4.9%) |
| *Medical & Surgical - Respiratory System* | 4.71% (4.5%, 4.9%) | 3.01% (2.9%, 3.2%) | 0.21% (0.05%, 0.66%) | 17.42% (17%, 18%) |
| *Medical & Surgical - Mouth and Throat* | 1.37% (1.3%, 1.5%) | 1.34% (1.2%, 1.4%) | 8.07% (6.7%, 9.6%) | 0.22% (0.13%, 0.37%) |
| *Medical & Surgical - Gastrointestinal System* | 12.90% (13%, 13%) | 13.26% (13%, 14%) | 0.14% (0.02%, 0.56%) | 13.10% (12%, 14%) |
| *Medical & Surgical - Hepatobiliary System and Pancreas* | 7.78% (7.6%, 8.0%) | 8.24% (8.0%, 8.5%) | 0.07% (0.00%, 0.45%) | 6.25% (5.7%, 6.9%) |
| *Medical & Surgical - Endocrine System* | 5.06% (4.9%, 5.2%) | 5.57% (5.4%, 5.8%) | 0.07% (0.00%, 0.45%) | 2.57% (2.2%, 3.0%) |
| *Medical & Surgical - Skin and Breast* | 9.81% (9.6%, 10%) | 10.88% (11%, 11%) | 3.45% (2.6%, 4.6%) | 3.77% (3.3%, 4.3%) |
| *Medical & Surgical - Subcutaneous Tissue and Fascia* | 0.91% (0.84%, 1.0%) | 1.03% (0.95%, 1.1%) | 0.55% (0.26%, 1.1%) | 0.16% (0.08%, 0.29%) |
| *Medical & Surgical - Muscles* | 0.20% (0.16%, 0.24%) | 0.23% (0.19%, 0.27%) | 0.14% (0.02%, 0.56%) | 0.00% (0.00%, 0.07%) |
| *Medical & Surgical - Tendons* | 0.93% (0.85%, 1.0%) | 1.09% (1.0%, 1.2%) | 0.21% (0.05%, 0.66%) | 0.01% (0.00%, 0.09%) |
| *Medical & Surgical - Bursae and Ligaments* | 0.15% (0.12%, 0.19%) | 0.16% (0.13%, 0.21%) | 0.00% (0.00%, 0.33%) | 0.07% (0.03%, 0.18%) |
| *Medical & Surgical - Head and Facial Bones* | 0.79% (0.72%, 0.87%) | 0.70% (0.63%, 0.78%) | 2.21% (1.5%, 3.1%) | 1.09% (0.86%, 1.4%) |
| *Medical & Surgical - Upper Bones* | 3.13% (3.0%, 3.3%) | 3.33% (3.2%, 3.5%) | 5.80% (4.7%, 7.2%) | 1.16% (0.93%, 1.4%) |
| *Medical & Surgical - Lower Bones* | 0.60% (0.54%, 0.67%) | 0.68% (0.61%, 0.76%) | 0.07% (0.00%, 0.45%) | 0.22% (0.13%, 0.37%) |
| *Medical & Surgical - Upper Joints* | 2.92% (2.8%, 3.1%) | 3.18% (3.0%, 3.3%) | 0.48% (0.21%, 1.0%) | 1.62% (1.3%, 2.0%) |
| *Medical & Surgical - Lower Joints* | 1.74% (1.6%, 1.9%) | 1.90% (1.8%, 2.0%) | 0.07% (0.00%, 0.45%) | 0.97% (0.76%, 1.2%) |
| *Medical & Surgical - Urinary System* | 5.86% (5.7%, 6.1%) | 6.57% (6.3%, 6.8%) | 0.76% (0.40%, 1.4%) | 2.04% (1.7%, 2.4%) |
| *Medical & Surgical - Female Reproductive System* | 6.10% (5.9%, 6.3%) | 6.03% (5.8%, 6.2%) | 35.68% (33%, 38%) | 0.39% (0.26%, 0.58%) |
| *Medical & Surgical - Male Reproductive System* | 3.50% (3.3%, 3.7%) | 4.05% (3.9%, 4.2%) | 0.35% (0.13%, 0.85%) | 0.30% (0.19%, 0.47%) |
| *Medical & Surgical - Anatomical Regions, General* | 4.70% (4.5%, 4.9%) | 5.09% (4.9%, 5.3%) | 0.48% (0.21%, 1.0%) | 2.86% (2.5%, 3.3%) |
| *Medical & Surgical - Anatomical Regions, Upper Extremities* | 0.26% (0.22%, 0.31%) | 0.30% (0.26%, 0.36%) | 0.07% (0.00%, 0.45%) | 0.01% (0.00%, 0.09%) |
| *Medical & Surgical - Anatomical regions, Lower Extremities* | 0.44% (0.39%, 0.50%) | 0.49% (0.43%, 0.55%) | 0.00% (0.00%, 0.33%) | 0.20% (0.12%, 0.35%) |
| *Obstetrics - Pregnancy* | 0.46% (0.41%, 0.52%) | 0.49% (0.43%, 0.56%) | 0.00% (0.00%, 0.33%) | 0.38% (0.25%, 0.56%) |
| *% (CI). Abbreviation: CI = Confidence Interval. | | | | |
|  | | | | |

Table S1. Medical and surgical metrics for overall cohort and stratified by three outcomes (>24 hour, ≤ 24hr and ICU). Values are reported as the percentage (CI). 95% confidence intervals shown in parentheses. **recovery time is defined as the time in hours from the end of operation to discharge.

DISTRIBUTION STATEMENT A. Approved for public release. Distribution is unlimited.

This material is based upon work supported by the Department of the Air Force under Air Force Contract No. FA8702-15-D-0001 or FA8702-25-D-B002. Any opinions, findings, conclusions or recommendations expressed in this material are those of the author(s) and do not necessarily reflect the views of the Department of the Air Force.

© 2026 Massachusetts Institute of Technology.

Delivered to the U.S. Government with Unlimited Rights, as defined in DFARS Part 252.227-7013 or 7014 (Feb 2014). Notwithstanding any copyright notice, U.S. Government rights in this work are defined by DFARS 252.227-7013 or DFARS 252.227-7014 as detailed above. Use of this work other than as specifically authorized by the U.S. Government may violate any copyrights that exist in this work.
