# Supplementary material for: Healthcare-Based Multimodal Recovery Prediction for the Warfighter: A Retrospective Study
Source: Mil Med. 2026 Aug 6;191(Suppl 1):631–40. doi: 10.1093/milmed/usag199 (PMC13445627; doi:10.1093/milmed/usag199)
Supplement: Supplementary file 1 [file usag199_supplementary_data.zip › usag199_Supplementary_Data/Table_S2.docx]

| **Table S2. Preoperative Signals** | | | | |
| --- | --- | --- | --- | --- |
| **Characteristic** | **Overall**  N = 56,226 (**95% CI**)* | **> 24 hr**  N = 47,882 (**95% CI**)* | **≤ 24 hr**  N = 1,449 (**95% CI**)* | **ICU**  N = 6,895 (**95% CI**)* |
| **Albumin, g/dL** | 4.17, 0.39 (4.2, 4.2) | 4.19, 0.37 (4.2, 4.2) | 4.32, 0.28 (4.3, 4.3) | 3.96, 0.51 (4.0, 4.0) |
| **Alanine transaminase, IU/L** | 22.32, 18.09 (22, 22) | 21.92, 17.17 (22, 22) | 21.83, 19.65 (21, 23) | 25.19, 23.05 (25, 26) |
| **Activated partial thromboplastin time, sec** | 31.48, 3.79 (31, 32) | 31.41, 3.44 (31, 31) | 31.50, 3.15 (31, 32) | 31.95, 5.74 (32, 32) |
| **Aspartate transaminase, IU/L** | 22.98, 12.80 (23, 23) | 22.52, 11.52 (22, 23) | 22.03, 11.94 (21, 23) | 26.33, 19.28 (26, 27) |
| **Total bilirubin, mg/dL** | 0.72, 0.45 (0.71, 0.72) | 0.71, 0.37 (0.70, 0.71) | 0.73, 0.35 (0.71, 0.75) | 0.80, 0.80 (0.78, 0.82) |
| **Calcium, mg/dL** | 9.12, 0.46 (9.1, 9.1) | 9.14, 0.44 (9.1, 9.1) | 9.21, 0.40 (9.2, 9.2) | 8.97, 0.54 (9.0, 9.0) |
| **Creatinine, mg/dL** | 0.86, 0.54 (0.86, 0.86) | 0.85, 0.50 (0.84, 0.85) | 0.81, 0.45 (0.79, 0.84) | 0.97, 0.75 (0.95, 0.99) |
| **High-sensitive C-reactive protein, mg/dL** | 0.48, 1.90 (0.47, 0.50) | 0.41, 1.66 (0.39, 0.42) | 0.16, 0.65 (0.13, 0.19) | 1.07, 3.13 (0.99, 1.1) |
| **Fibrinogen, mg/dL** | 310.79, 67.07 (310, 311) | 309.82, 63.74 (309, 310) | 292.72, 42.01 (291, 295) | 321.30, 88.95 (319, 323) |
| **Glucose, mg/dL** | 108.61, 27.38 (108, 109) | 107.23, 25.53 (107, 107) | 103.07, 22.74 (102, 104) | 119.32, 36.65 (118, 120) |
| **Hemoglobin, g/dL** | 13.20, 1.62 (13, 13) | 13.25, 1.59 (13, 13) | 13.43, 1.58 (13, 14) | 12.83, 1.77 (13, 13) |
| **Hematocrit, percentage** | 39.47, 4.41 (39, 40) | 39.62, 4.30 (40, 40) | 40.31, 4.11 (40, 41) | 38.25, 4.99 (38, 38) |
| **Lymphocyte, percentage** | 30.81, 9.22 (31, 31) | 31.24, 8.92 (31, 31) | 32.69, 8.22 (32, 33) | 27.41, 10.63 (27, 28) |
| **Platelet count, /nL** | 242.88, 66.94 (242, 243) | 244.43, 65.05 (244, 245) | 252.53, 62.74 (249, 256) | 230.04, 78.24 (228, 232) |
| **Potassium, mmol/L** | 4.15, 0.38 (4.1, 4.2) | 4.15, 0.38 (4.1, 4.2) | 4.24, 0.38 (4.2, 4.3) | 4.11, 0.42 (4.1, 4.1) |
| **Prothrombin time, international normalized ratio** | 0.99, 0.10 (0.99, 0.99) | 0.99, 0.08 (0.99, 0.99) | 0.99, 0.07 (0.98, 0.99) | 1.03, 0.19 (1.0, 1.0) |
| **Segmented neutrophil, percentage** | 58.62, 10.17 (59, 59) | 58.26, 9.86 (58, 58) | 57.06, 9.27 (57, 58) | 61.51, 11.84 (61, 62) |
| **Sodium, mmol/L** | 139.53, 2.36 (140, 140) | 139.61, 2.25 (140, 140) | 139.58, 2.01 (139, 140) | 138.93, 2.99 (139, 139) |
| **Total Protein, g/dL** | 7.07, 0.54 (7.1, 7.1) | 7.09, 0.51 (7.1, 7.1) | 7.24, 0.41 (7.2, 7.3) | 6.87, 0.66 (6.9, 6.9) |
| **White blood cell count, /nL** | 6.51, 2.17 (6.5, 6.5) | 6.44, 2.06 (6.4, 6.5) | 6.47, 1.96 (6.4, 6.6) | 7.01, 2.79 (6.9, 7.1) |
| **Maximum body temperature, °C** | 36.77, 0.41 (37, 37) | 36.75, 0.40 (37, 37) | 36.53, 0.40 (37, 37) | 36.94, 0.47 (37, 37) |
| **Mean body temperature, °C** | 36.38, 0.25 (36, 36) | 36.37, 0.24 (36, 36) | 36.33, 0.26 (36, 36) | 36.43, 0.28 (36, 36) |
| **Minimum body temperature, °C** | 36.06, 0.20 (36, 36) | 36.06, 0.19 (36, 36) | 36.16, 0.24 (36, 36) | 36.02, 0.22 (36, 36) |
| **Use of hemodialysis or continuous renal replacement therapy** | 0.05% (0.04%, 0.08%) | 0.01% (0.00%, 0.03%) | 0.07% (0.00%, 0.45%) | 0.33% (0.22%, 0.51%) |
| **Use of extracorporeal membrane oxygenator** | 0.02% (0.01%, 0.04%) | 0.00% (0.00%, 0.02%) | 0.00% (0.00%, 0.33%) | 0.13% (0.06%, 0.26%) |
| **Maximum heart rate, /min** | 86.79, 16.63 (87, 87) | 86.25, 16.58 (86, 86) | 84.59, 17.75 (84, 86) | 90.98, 16.08 (91, 91) |
| **Mean heart rate, /min** | 72.40, 10.99 (72, 72) | 72.15, 10.80 (72, 72) | 74.06, 12.02 (73, 75) | 73.77, 11.88 (73, 74) |
| **Minimum heart rate, /min** | 61.35, 9.35 (61, 61) | 61.31, 9.14 (61, 61) | 65.55, 11.52 (65, 66) | 60.77, 9.97 (61, 61) |
| **Existence of any parameter related to use of Intra-aortic balloon pump** | 0.02% (0.01%, 0.04%) | 0.01% (0.00%, 0.02%) | 0.00% (0.00%, 0.33%) | 0.15% (0.07%, 0.28%) |
| **Maximum non-invasive diastolic blood pressure, mmHg** | 83.09, 9.46 (83, 83) | 82.88, 9.39 (83, 83) | 79.43, 10.11 (79, 80) | 85.38, 9.41 (85, 86) |
| **Mean non-invasive diastolic blood pressure, mmHg** | 74.04, 8.59 (74, 74) | 74.19, 8.54 (74, 74) | 74.65, 9.60 (74, 75) | 72.93, 8.57 (73, 73) |
| **Minimum non-invasive diastolic blood pressure, mmHg** | 64.35, 10.26 (64, 64) | 64.78, 10.08 (65, 65) | 69.57, 11.43 (69, 70) | 60.21, 10.11 (60, 60) |
| **Maximum non-invasive systolic blood pressure, mmHg** | 134.17, 16.74 (134, 134) | 133.51, 16.44 (133, 134) | 127.15, 16.66 (126, 128) | 140.23, 17.29 (140, 141) |
| **Mean non-invasive systolic blood pressure, mmHg** | 120.53, 12.91 (120, 121) | 120.45, 12.81 (120, 121) | 120.13, 13.84 (119, 121) | 121.21, 13.40 (121, 122) |
| **Minimum non-invasive systolic blood pressure, mmHg** | 107.82, 12.71 (108, 108) | 108.15, 12.52 (108, 108) | 113.49, 14.77 (113, 114) | 104.34, 12.71 (104, 105) |
| **Maximum respiration rate, /min** | 18.73, 1.89 (19, 19) | 18.61, 1.62 (19, 19) | 17.93, 1.53 (18, 18) | 19.71, 3.04 (20, 20) |
| **Mean respiration rate, /min** | 17.95, 0.94 (18, 18) | 17.92, 0.89 (18, 18) | 17.48, 1.14 (17, 18) | 18.24, 1.15 (18, 18) |
| **Minimum respiration rate, /min** | 17.19, 1.24 (17, 17) | 17.24, 1.17 (17, 17) | 17.07, 1.22 (17, 17) | 16.89, 1.63 (17, 17) |
| **Use of mechanical ventilation** | 0.14% (0.11%, 0.17%) | 0.06% (0.04%, 0.08%) | 0.00% (0.00%, 0.33%) | 0.73% (0.54%, 0.96%) |
| *% (CI) or Mean, SD (CI). Abbreviation: ,CI = Confidence Interval, SD = standard deviation | | | | |
|  | | | | |

**Table S2. Preoperative signals (vital signs and laboratory values) for overall cohort and stratified by three outcomes (>24 hour, ≤ 24hr and ICU). Values are reported as the mean, SD (CI) for continuous variables, and as percentage (CI) for categorical and binary variables. 95% confidence intervals shown in parentheses.**

DISTRIBUTION STATEMENT A. Approved for public release. Distribution is unlimited.

This material is based upon work supported by the Department of the Air Force under Air Force Contract No. FA8702-15-D-0001 or FA8702-25-D-B002. Any opinions, findings, conclusions or recommendations expressed in this material are those of the author(s) and do not necessarily reflect the views of the Department of the Air Force.

© 2026 Massachusetts Institute of Technology.

Delivered to the U.S. Government with Unlimited Rights, as defined in DFARS Part 252.227-7013 or 7014 (Feb 2014). Notwithstanding any copyright notice, U.S. Government rights in this work are defined by DFARS 252.227-7013 or DFARS 252.227-7014 as detailed above. Use of this work other than as specifically authorized by the U.S. Government may violate any copyrights that exist in this work.
