# Supplementary material for: Healthcare-Based Multimodal Recovery Prediction for the Warfighter: A Retrospective Study
Source: Mil Med. 2026 Aug 6;191(Suppl 1):631–40. doi: 10.1093/milmed/usag199 (PMC13445627; doi:10.1093/milmed/usag199)
Supplement: Supplementary file 1 [file usag199_supplementary_data.zip › usag199_Supplementary_Data/Table_S3.docx]

| **Table S3. Intraoperative Vital Signs** | | | | |
| --- | --- | --- | --- | --- |
| **Characteristic** | **Overall**  N = 56,226 (**95% CI**)* | **> 24 hr**  N = 47,882 (**95% CI**)* | **≤ 24 hr**  N = 1,449 (**95% CI**)* | **ICU**  N = 6,895 (**95% CI**)* |
| **Infused dose of alfentanil** | 0.00% (0.00%, 0.01%) | 0.00% (0.00%, 0.01%) | 0.00% (0.00%, 0.33%) | 0.00% (0.00%, 0.07%) |
| **Infused volume of 20% albumin** | 0.91% (0.84%, 1.0%) | 0.15% (0.12%, 0.20%) | 0.00% (0.00%, 0.33%) | 6.38% (5.8%, 7.0%) |
| **Infused volume of 5% albumin** | 0.06% (0.04%, 0.09%) | 0.02% (0.01%, 0.04%) | 0.00% (0.00%, 0.33%) | 0.36% (0.24%, 0.54%) |
| **Maximum arterial diastolic blood pressure, mmHg** | 80.99, 6.17 (81, 81) | 81.44, 5.51 (81, 81) | 81.76, 2.47 (82, 82) | 77.70, 9.28 (77, 78) |
| **Mean arterial diastolic blood pressure, mmHg** | 61.03, 5.18 (61, 61) | 61.45, 4.80 (61, 61) | 60.80, 1.65 (61, 61) | 58.14, 6.93 (58, 58) |
| **Minimum arterial diastolic blood pressure, mmHg** | 45.12, 4.52 (45, 45) | 45.27, 4.48 (45, 45) | 44.11, 1.61 (44, 44) | 44.29, 5.04 (44, 44) |
| **Maximum arterial mean blood pressure, mmHg** | 105.45, 8.63 (105, 106) | 105.76, 8.36 (106, 106) | 105.58, 5.38 (105, 106) | 103.26, 10.52 (103, 104) |
| **Mean arterial mean blood pressure, mmHg** | 78.19, 7.60 (78, 78) | 78.63, 7.35 (79, 79) | 78.06, 4.40 (78, 78) | 75.17, 9.02 (75, 75) |
| **Minimum arterial mean blood pressure, mmHg** | 44.58, 20.28 (44, 45) | 45.80, 19.26 (46, 46) | 51.53, 5.95 (51, 52) | 34.66, 25.49 (34, 35) |
| **Maximum arterial systolic blood pressure, mmHg** | 149.21, 9.19 (149, 149) | 149.43, 8.57 (149, 150) | 149.97, 2.47 (150, 150) | 147.53, 13.19 (147, 148) |
| **Mean arterial systolic blood pressure, mmHg** | 114.34, 7.77 (114, 114) | 114.74, 7.08 (115, 115) | 114.21, 2.93 (114, 114) | 111.59, 11.55 (111, 112) |
| **Minimum arterial systolic blood pressure, mmHg** | 83.32, 8.29 (83, 83) | 83.54, 8.00 (83, 84) | 82.35, 4.15 (82, 83) | 81.99, 10.48 (82, 82) |
| **Maximum bispectral index** | 51.82, 7.38 (52, 52) | 51.84, 7.46 (52, 52) | 50.30, 7.51 (50, 51) | 51.99, 6.77 (52, 52) |
| **Mean bispectral index** | 40.74, 5.34 (41, 41) | 40.85, 5.39 (41, 41) | 40.97, 5.51 (41, 41) | 39.94, 4.87 (40, 40) |
| **Minimum bispectral index** | 32.92, 5.80 (33, 33) | 33.12, 5.81 (33, 33) | 34.47, 6.31 (34, 35) | 31.26, 5.33 (31, 31) |
| **Maximum body temperature, °C** | 36.11, 0.93 (36, 36) | 36.10, 0.91 (36, 36) | 35.71, 1.57 (36, 36) | 36.24, 0.79 (36, 36) |
| **Mean body temperature, °C** | 34.93, 2.08 (35, 35) | 34.96, 2.08 (35, 35) | 34.56, 2.44 (34, 35) | 34.79, 2.03 (35, 35) |
| **Minimum body temperature, °C** | 31.32, 5.78 (31, 31) | 31.39, 5.79 (31, 31) | 31.95, 5.37 (32, 32) | 30.73, 5.81 (31, 31) |
| **Transfused cryoprecipitate** | 0.36% (0.31%, 0.41%) | 0.04% (0.02%, 0.06%) | 0.00% (0.00%, 0.33%) | 2.65% (2.3%, 3.1%) |
| **Dobutamine use** | 1.07% (0.98%, 1.2%) | 0.05% (0.04%, 0.08%) | 0.00% (0.00%, 0.33%) | 8.32% (7.7%, 9.0%) |
| **Dopamine use** | 0.42% (0.37%, 0.48%) | 0.14% (0.11%, 0.18%) | 0.07% (0.00%, 0.45%) | 2.42% (2.1%, 2.8%) |
| **Injected dose of Ephedrine** | 38.15% (38%, 39%) | 38.38% (38%, 39%) | 23.05% (21%, 25%) | 39.74% (39%, 41%) |
| **Injected dose of Epinephrine** | 0.33% (0.28%, 0.38%) | 0.01% (0.00%, 0.03%) | 0.00% (0.00%, 0.33%) | 2.61% (2.3%, 3.0%) |
| **Epinephrine continuous infusion (binary)** | 0.19% (0.16%, 0.23%) | 0.01% (0.00%, 0.02%) | 0.07% (0.00%, 0.45%) | 1.48% (1.2%, 1.8%) |
| **Desflurane use** | 21.85% (22%, 22%) | 23.69% (23%, 24%) | 29.12% (27%, 32%) | 7.54% (6.9%, 8.2%) |
| **Anesthetic agent use** | 21.85% (22%, 22%) | 23.69% (23%, 24%) | 29.12% (27%, 32%) | 7.54% (6.9%, 8.2%) |
| **Isoflurane use** | 0.00% (0.00%, 0.01%) | 0.00% (0.00%, 0.01%) | 0.00% (0.00%, 0.33%) | 0.00% (0.00%, 0.07%) |
| **Sevoflurane use** | 34.33% (34%, 35%) | 36.15% (36%, 37%) | 32.23% (30%, 35%) | 22.13% (21%, 23%) |
| **Transfused fresh frozen plasma** | 1.89% (1.8%, 2.0%) | 0.37% (0.31%, 0.42%) | 0.14% (0.02%, 0.56%) | 12.85% (12%, 14%) |
| **Maximum heart rate, /min** | 92.24, 13.35 (92, 92) | 92.24, 13.29 (92, 92) | 90.93, 14.12 (90, 92) | 92.55, 13.58 (92, 93) |
| **Mean heart rate, /min** | 70.32, 11.04 (70, 70) | 70.04, 10.81 (70, 70) | 73.16, 11.28 (73, 74) | 71.63, 12.34 (71, 72) |
| **Minimum heart rate, /min** | 57.72, 9.79 (58, 58) | 57.82, 9.52 (58, 58) | 61.34, 10.44 (61, 62) | 56.27, 11.11 (56, 57) |
| **Injected dose of midazolam** | 2.72% (2.6%, 2.9%) | 0.67% (0.60%, 0.75%) | 0.28% (0.09%, 0.76%) | 17.45% (17%, 18%) |
| **Minute maximum ventilation, L/min** | 6.75, 1.47 (6.7, 6.8) | 6.75, 1.48 (6.7, 6.8) | 6.60, 1.50 (6.5, 6.7) | 6.74, 1.36 (6.7, 6.8) |
| **Minute mean ventilation, L/min** | 5.12, 1.18 (5.1, 5.1) | 5.14, 1.19 (5.1, 5.2) | 5.04, 1.14 (5.0, 5.1) | 4.97, 1.06 (4.9, 5.0) |
| **Minute minimum ventilation, L/min** | 2.16, 1.27 (2.2, 2.2) | 2.20, 1.29 (2.2, 2.2) | 2.27, 1.35 (2.2, 2.3) | 1.84, 1.06 (1.8, 1.9) |
| **Milrinone use** | 0.28% (0.24%, 0.33%) | 0.20% (0.16%, 0.24%) | 0.00% (0.00%, 0.33%) | 0.94% (0.73%, 1.2%) |
| **Nitrous oxide (binary)** | 1.16% (1.1%, 1.3%) | 1.25% (1.2%, 1.4%) | 0.97% (0.55%, 1.7%) | 0.59% (0.43%, 0.81%) |
| **Norepinephrine use** | 2.10% (2.0%, 2.2%) | 0.42% (0.36%, 0.48%) | 0.21% (0.05%, 0.66%) | 14.20% (13%, 15%) |
| **Maximum non-invasive diastolic blood pressure, mmHg** | 85.22, 10.55 (85, 85) | 85.66, 10.42 (86, 86) | 83.28, 11.61 (83, 84) | 82.56, 10.76 (82, 83) |
| **Mean non-invasive diastolic blood pressure, mmHg** | 68.24, 9.51 (68, 68) | 68.08, 9.39 (68, 68) | 65.66, 9.96 (65, 66) | 69.92, 9.98 (70, 70) |
| **Minimum non-invasive diastolic blood pressure, mmHg** | 55.49, 10.10 (55, 56) | 55.05, 9.66 (55, 55) | 54.02, 9.15 (54, 54) | 58.91, 12.39 (59, 59) |
| **Maximum non-invasive mean blood pressure, mmHg** | 110.22, 13.90 (110, 110) | 110.77, 13.72 (111, 111) | 107.48, 14.83 (107, 108) | 106.94, 14.41 (107, 107) |
| **Mean non-invasive mean blood pressure, mmHg** | 87.07, 11.52 (87, 87) | 86.82, 11.22 (87, 87) | 84.48, 11.78 (84, 85) | 89.35, 13.12 (89, 90) |
| **Minimum non-invasive mean blood pressure, mmHg** | 70.20, 12.48 (70, 70) | 69.58, 11.72 (69, 70) | 69.29, 10.84 (69, 70) | 74.65, 16.38 (74, 75) |
| **Maximum non-invasive systolic blood pressure, mmHg** | 151.78, 20.01 (152, 152) | 152.51, 19.75 (152, 153) | 146.10, 20.70 (145, 147) | 147.93, 20.99 (147, 148) |
| **Mean non-invasive systolic blood pressure, mmHg** | 120.23, 16.33 (120, 120) | 119.82, 15.80 (120, 120) | 116.82, 15.74 (116, 118) | 123.79, 19.28 (123, 124) |
| **Minimum non-invasive systolic blood pressure, mmHg** | 97.34, 17.51 (97, 97) | 96.45, 16.39 (96, 97) | 97.13, 14.34 (96, 98) | 103.62, 23.36 (103, 104) |
| **Nitroglycerin use** | 1.54% (1.4%, 1.6%) | 0.30% (0.26%, 0.36%) | 0.07% (0.00%, 0.45%) | 10.41% (9.7%, 11%) |
| **Transfused platelet concentrate (binary)** | 0.91% (0.83%, 0.99%) | 0.10% (0.08%, 0.14%) | 0.00% (0.00%, 0.33%) | 6.69% (6.1%, 7.3%) |
| **Positive end expiratory pressure (binary)** | 72.37% (72%, 73%) | 72.33% (72%, 73%) | 75.22% (73%, 77%) | 72.05% (71%, 73%) |
| **Phenylephrine continuous infusion** | 6.03% (5.8%, 6.2%) | 4.34% (4.2%, 4.5%) | 0.28% (0.09%, 0.76%) | 18.93% (18%, 20%) |
| **Injected dose of phenylephrine** | 12.48% (12%, 13%) | 9.05% (8.8%, 9.3%) | 1.86% (1.3%, 2.7%) | 38.48% (37%, 40%) |
| **Transfused platelet pheresis** | 0.05% (0.04%, 0.08%) | 0.01% (0.00%, 0.02%) | 0.00% (0.00%, 0.33%) | 0.38% (0.25%, 0.56%) |
| **Injected dose of propofol** | 44.54% (44%, 45%) | 47.79% (47%, 48%) | 65.70% (63%, 68%) | 17.48% (17%, 18%) |
| **Infused volume of plasma solution** | 49.94% (50%, 50%) | 46.32% (46%, 47%) | 17.46% (16%, 20%) | 81.89% (81%, 83%) |
| **Transfused red blood cell** | 6.93% (6.7%, 7.1%) | 3.96% (3.8%, 4.1%) | 0.14% (0.02%, 0.56%) | 28.96% (28%, 30%) |
| **Remifentanil use** | 24.01% (24%, 24%) | 22.61% (22%, 23%) | 8.42% (7.1%, 10%) | 37.01% (36%, 38%) |
| **Maximum respiration rate, /min** | 16.74, 2.94 (17, 17) | 16.78, 2.93 (17, 17) | 16.10, 3.04 (16, 16) | 16.56, 2.95 (16, 17) |
| **Mean respiration rate, /min** | 12.14, 1.84 (12, 12) | 12.14, 1.84 (12, 12) | 12.16, 1.78 (12, 12) | 12.08, 1.84 (12, 12) |
| **Minimum respiration rate, /min** | 6.98, 3.27 (6.9, 7.0) | 7.06, 3.27 (7.0, 7.1) | 7.20, 3.57 (7.0, 7.4) | 6.35, 3.11 (6.3, 6.4) |
| **Injected dose of sufentanil,** | 1.20% (1.1%, 1.3%) | 0.05% (0.03%, 0.08%) | 0.00% (0.00%, 0.33%) | 9.41% (8.7%, 10%) |
| **Maximum peripheral oxygen saturation, percentage** |  |  |  |  |
| *85* | 0.00% (0.00%, 0.01%) | 0.00% (0.00%, 0.01%) | 0.00% (0.00%, 0.33%) | 0.00% (0.00%, 0.07%) |
| *95* | 0.02% (0.01%, 0.03%) | 0.01% (0.01%, 0.03%) | 0.00% (0.00%, 0.33%) | 0.06% (0.02%, 0.16%) |
| *98* | 0.07% (0.05%, 0.10%) | 0.06% (0.04%, 0.09%) | 0.28% (0.09%, 0.76%) | 0.10% (0.04%, 0.22%) |
| *99* | 1.17% (1.1%, 1.3%) | 1.21% (1.1%, 1.3%) | 3.24% (2.4%, 4.3%) | 0.49% (0.35%, 0.70%) |
| *100* | 98.74% (99%, 99%) | 98.72% (99%, 99%) | 96.48% (95%, 97%) | 99.35% (99%, 100%) |
| **Mean peripheral oxygen saturation, percentage** | 99.11, 1.04 (99, 99) | 99.11, 1.03 (99, 99) | 98.79, 1.38 (99, 99) | 99.22, 0.96 (99, 99) |
| **Minimum peripheral oxygen saturation, percentage** |  |  |  |  |
| *85* | 33.03% (33%, 33%) | 32.47% (32%, 33%) | 26.43% (24%, 29%) | 38.30% (37%, 39%) |
| *95* | 32.56% (32%, 33%) | 32.22% (32%, 33%) | 26.02% (24%, 28%) | 36.32% (35%, 37%) |
| *98* | 10.84% (11%, 11%) | 10.91% (11%, 11%) | 14.15% (12%, 16%) | 9.66% (9.0%, 10%) |
| *99* | 13.35% (13%, 14%) | 13.75% (13%, 14%) | 18.22% (16%, 20%) | 9.49% (8.8%, 10%) |
| *100* | 10.22% (10%, 10%) | 10.65% (10%, 11%) | 15.18% (13%, 17%) | 6.24% (5.7%, 6.8%) |
| **Injected dose of vasopressin** | 0.20% (0.16%, 0.24%) | 0.00% (0.00%, 0.02%) | 0.00% (0.00%, 0.33%) | 1.58% (1.3%, 1.9%) |
| **Maximum tidal volume, ml** | 500.41, 85.18 (500, 501) | 500.10, 85.64 (499, 501) | 489.65, 84.51 (485, 494) | 504.82, 81.79 (503, 507) |
| **Mean tidal volume, ml** | 430.51, 73.40 (430, 431) | 432.32, 73.06 (432, 433) | 417.96, 68.57 (414, 421) | 420.53, 75.66 (419, 422) |
| **Minimum tidal volume, ml** | 231.09, 90.09 (230, 232) | 232.38, 90.41 (232, 233) | 239.89, 94.75 (235, 245) | 220.30, 85.97 (218, 222) |
| * % (CI) or Mean, SD (CI). Abbreviation: CI = Confidence Interval, SD = standard deviation. | | | | |
|  | | | | |

**Table S3. Intraoperative vital signs for overall cohort and stratified by three outcomes (>24 hour, ≤ 24hr and ICU). Values are reported as the mean, SD (CI) for continuous variables, and as percentage (CI) for categorical and binary variables. 95% confidence intervals shown in parentheses.**

DISTRIBUTION STATEMENT A. Approved for public release. Distribution is unlimited.

This material is based upon work supported by the Department of the Air Force under Air Force Contract No. FA8702-15-D-0001 or FA8702-25-D-B002. Any opinions, findings, conclusions or recommendations expressed in this material are those of the author(s) and do not necessarily reflect the views of the Department of the Air Force.

© 2026 Massachusetts Institute of Technology.

Delivered to the U.S. Government with Unlimited Rights, as defined in DFARS Part 252.227-7013 or 7014 (Feb 2014). Notwithstanding any copyright notice, U.S. Government rights in this work are defined by DFARS 252.227-7013 or DFARS 252.227-7014 as detailed above. Use of this work other than as specifically authorized by the U.S. Government may violate any copyrights that exist in this work.
